# Most of the Northern Hemisphere Permafrost Remains under Climate Change

**DOI:** 10.1038/s41598-019-39942-4

**Published:** 2019-03-01

**Authors:** Chenghai Wang, Zhilan Wang, Ying Kong, Feimin Zhang, Kai Yang, Tingjun Zhang

**Affiliations:** Key Laboratory of Arid Climate Change and Disaster Reduction of Gansu Province, School of Atmospheric Sciences, University Corporation for Polar Research, Lanzhou University, Lanzhou, 730000 China

## Abstract

Degradation of cryospheric components such as arctic sea ice and permafrost may pose a threat to the Earth’s climate system. A rise of 2 °C above pre-industrial global surface temperature is considered to be a risk-level threshold. This study investigates the impacts of global temperature rises of 1.5 °C and 2 °C on the extent of the permafrost in the Northern Hemisphere (NH), based on the 17 models of Coupled Model Intercomparison Project Phase 5 (CMIP5). Results show that, when global surface temperature rises by 1.5 °C, the average permafrost extent projected under Representative Concentration Pathway (RCP) scenarios would decrease by 23.58% for RCP2.6 (2027–2036), 24.1% for RCP4.5 (2026–2035) and 25.55% for RCP8.5 (2023–2032). However, uncertainty in the results persists because of distinct discrepancies among the models. When the global surface temperature rises by 2 °C, about one-third of the permafrost would disappear; in other words, most of the NH permafrost would still remain even in the RCP8.5 (2037–2046) scenario. The results of the study highlight that the NH permafrost might be able to stably exist owing to its relatively slow degradation. This outlook gives reason for hope for future maintenance and balance of the cryosphere and climate systems.

## Introduction

Frozen ground is composed of various ice-rich soils and rocks, which are the products of lithosphere–soil–atmosphere interactions during the process of material exchange. Permafrost is a key component of the cryosphere, which occupies around a quarter of the Earth’s land area in the Northern Hemisphere^1,2^. Because of its high sensitivity to, and feedback relationship with, the climate, permafrost plays a critical role as an indicator of global climate change^3–5^. As the global surface temperature has continued to rise over the recent decades^6,7^, the risk of permafrost degradation has also increased. The degradation of the permafrost may affect the climate system via many factors such as local ecological balance, hydrological processes, energy exchange and the carbon cycle, as well as the engineering infrastructure in cold regions and even extreme weather events^8–17^.

Over the last 100 years, global climate has warmed distinctly, with the global surface temperature increasing by 0.74 °C ± 0.18 °C between 1906 and 2005^4^. In particular, over the past 30 years, the Earth has experienced a period of rapid warming. The increase in observed global average surface temperature between 1880 and 2012 was 0.85 (0.65–1.06) °C^18^. However, the magnitude of warming shows remarkable regional differences. Regions of significant warming include the high latitudes of the NH, including Arctic and high-elevation areas such as the Qinghai–Tibet Plateau. Warming in these areas is the most significant over this period, reaching up to 2.5 °C^16,19–21^. Global warming is naturally amplified in the Arctic, where a slight increase in mean annual air temperature can result in a change of state for large regions of frozen ground, in terms of both active layer thickness and area^8,11^.

Global warming may lead to a reduction in the extent and volume of the cryosphere, which will, in turn, have a positive feedback effect on warming^22,23^. Under the influence of global warming, permafrost temperature has evidently shown an increasing trend since 1980 in most parts of the NH^24^. However, because of the spatial differences found in climate change itself, as well as the heterogeneity of soil properties, there remain regional differences in soil temperatures, permafrost boundaries and active layer thicknesses (ALT)^25^. A number of studies have reported these regional variations in the effects of global warming on the permafrost layer. For example, the temperature at a depth of 20 m in the permafrost has increased at a rate of 0.13 °C/a in northern Alaska since the 1980s^26^. The temperature at a depth of 6 m in the permafrost increased by an amount between 0.12 °C and 0.67 °C on the high-altitude Qinghai–Tibet Plateau over the period 1996–2006^27^. In northern Europe, permafrost temperature increased by an amount between 0.3 °C and 2 °C between 1971 and 2010^28^. Degradation of the permafrost has also been observed in many other regions, especially in Siberia, Sweden, Canada and Alaska^29,30^. In Russia’s Vorkuta region, a permafrost layer 10 to 15 m thick melted completely between 1975 and 2005, and the southern limit of the permafrost shifted 80 km northwards^28^. ALT deepened by approximately 20 cm in Russia between 1956 and 1990^31^. The lower altitudinal limit of mountain permafrost has risen by 25 m over the last 30 years at Xidatan in the interior of the Qinghai–Tibet Plateau^3,32^.

The ongoing increase in greenhouse gas (GHG) emissions is resulting in frequent occurrences of extreme weather and climate events, glacier retreat, sea level rise and deteriorating human health. Therefore, a 2 °C increase in global surface temperature from pre-industrial levels is considered to be a risk-level threshold^33,34^. Consequently, if the rise in global surface temperature cannot be restricted to 2 °C, human society will probably face major climate-related disasters induced by global warming^35^. The Paris Climate Agreement (2015) proposed a goal of reducing greenhouse gas levels and emphasised that global mean surface temperature increases should be restricted to 2 °C, or even 1.5 °C, compared to the pre-industrial level. Predicting the response of various components of the Earth’s system to a temperature rise of 2 °C has received much attention^36–42^. As a modulator of the Earth’s climate, the cryosphere is a particularly crucial component. Anisimov and Nelson^8^ investigated permafrost distribution in the NH using a frost index under a global mean temperature rise of 2 °C and doubling of CO_2_ levels. Their projected permafrost extent was 18.34 × 10^6^, 18.74 × 10^6^ and 19.17 × 10^6^ km^2^, respectively, as obtained using the Geophysical Fluid Dynamics Laboratory (GFDL) model, the Goddard Institute for Space Studies (GISS) model and the United Kingdom Meteorological Office (UKMO) model. The near-surface permafrost area in the NH has been estimated using Community Climate System Model Version 4 (CCSM4), revealing that, the permafrost areas during the 2030–2049 period would be 9.3 × 10^6^, 9.0 × 10^6^, 8.1 × 10^6^ and 8.7 × 10^6^ km^2^, respectively, under RCP2.6, RCP4.5, RCP6.0 and RCP8.5^43^. Slater and Lawence^30^ compared the permafrost extent estimated by soil temperature and surface frost index in Coupled Model Intercomparison Project Phase 5 (CMIP5) models. Their results show that permafrost estimation based on CMIP5 models has evident uncertainty, which is closely related to the different structure and parameterisations in different land surface models (LSMs). Furthermore, current models still have limitations in representing subgrid-scale permafrost distribution (e.g. sporadic and isolated permafrost) because of parameterisation uncertainty regarding the occurrence of soil freeze or thaw in LSMs^30^. Koven *et al*.^44^ suggested that the model differences can be traced to the differences in the coupling between either near-surface air and shallow soil temperatures or shallow and deeper (1 m) soil temperatures. Wang *et al*.^16^ suggested that the permafrost over the Qinghai–Tibet Plateau will significantly degrade in the Bayan Har Mountains and Tanggula Mountains by 2050 under the high-emission scenario. Although previous studies have demonstrated that the permafrost will degrade because of temperature rises caused by greenhouse gas (GHG) emissions, the likely extent of permafrost degradation under a particular risk-level threshold remains to be determined.

Previous studies have mainly focused on the permafrost projection in the 21^st^ century in different RCP scenarios. However, the relation between future permafrost extent and temperature rise at a specified threshold is still not fully addressed. This study focuses on what degree or how large in extent would the permafrost degradation response be to global warming under global temperature rise at risk-level thresholds of 1.5 °C, perhaps a maximum threshold of 2 °C. This is an important issue to the global community and is helpful for making adaptation and countermeasure policies.

To more objectively estimate permafrost distribution over the Northern Hemisphere, the Kudryavtsev method^45^ and the multi-model ensemble mean (MME) were applied to the 17 CMIP5 global climate models to project the variability of NH permafrost extent when surface temperatures reach thresholds of 1.5 °C and 2 °C above pre-industrial levels. The validation of the periods that correspond to thresholds of 1.5 °C and 2 °C is described in Section 2. Section 3 considers the permafrost area over the NH simulated by the CMIP5 models, including the present-day permafrost area and future permafrost area projected under the scenarios of 1.5 °C and 2 °C of temperature increase. Finally, Section 4 summarises key findings. The specific datasets and methods used in this study are described in the Methods section.

## Results

### Confirmation of the time taken to reach 1.5 °C and 2 °C thresholds

The determination of the period that corresponds to the thresholds of 1.5 °C and 2 °C rise in temperature is different under different models or different RCP scenarios (RCP4.5, 6.5 and 8.5). This determination is also closely dependent on the definition of the pre-industrial period. The periods 1861–1880, 1861–1900 and 1871–1900 have all been used as reference periods for the climate state in pre-industrial times in previous studies^38,39,46^. Considering the difference in start times for the historical simulations in the various models, the average of 1861–1880 was used as the reference period in the present study, which is consistent with the approach of the IPCC^20^. The MME indicates that the global average surface temperature during the pre-industrial period was approximately 14.2 °C. The data used for the analysis were the monthly surface temperature datasets from the 17 CMIP5 global climate models. Most of the models are able to reproduce the increase in surface temperature over the past 50 to 100 years^46^, and the correlation coefficient is greater than 0.71 between 12 such models and Climatic Research Unit (CRU) Time-Series (TS, version 3.21)^47^. Because surface temperature does not necessarily continue to immediately rise once it reaches a threshold, we define the year that the global mean surface temperature reaches the 1.5 °C or and 2.0 °C threshold as follows: (1) the first year that reaches the threshold in ascending order of time and (2) the first year that reaches the threshold and is maintained for five consecutive years, similar to the approach of Zhang *et al*.^46^ (Table 1).

**Figure 1:**
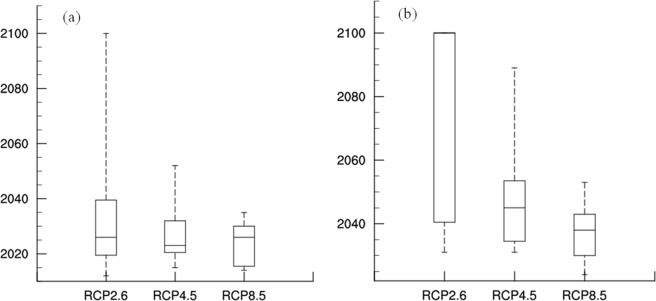
The time distribution of 17 models when global mean surface temperature rises to (**a**) 1.5 °C and (**b**) 2 °C above pre-industrial (1861–1880) under RCPs.

Figure 1 and Table 1 present the statistical results from individual models. The results indicate the time that the global mean surface temperature reaches the 1.5 °C and 2 °C thresholds. The results also clearly show that the simulation time in different RCP scenarios taken to reach the thresholds of 1.5 °C and 2 °C under different RCP scenarios are quite different from one another, especially for RCP2.6. Since each model in CMIP5 represents a possible future, some models (such as NorESM1-M in Table 1) are not excluded in this study. The results shown in Table 1 also show that over half of the models would not reach the 2 °C threshold in the 21^st^ century. Because the data in most of the RCPs cover the period of 2006–2100, years beyond 2100 are marked as 2100, which causes the maximum, median and upper quartiles to overlap. The results of the MME (Table 1) show that the threshold of 1.5 °C occurs in 2027, 2026 and 2023, respectively, under RCP2.6, RCP4.5 and RCP8.5. However, the global mean surface temperature under RCP2.6 does not reach the 2 °C threshold until the end of the 21^st^ century and only reaches about 1.9 °C by 2100. Under RCP4.5 and RCP8.5, the 2 °C threshold is reached in 2046 and 2037, respectively, which agrees with previous studies^25,34,39^. We note clear differences in the timing of the 2 °C threshold being reached under the three RCPs. For instance, the stronger the radiation force, the sooner the threshold of 2 °C is reached, although this relationship is not evident for the 1.5 °C threshold in these RCPs. A possible reason for this observation is that the present global mean surface temperature is close to the threshold of the 1.5 °C scenario, and radiation has a delayed effect on surface temperature. Consequently, there is little difference between radiative forcing and the year in which global average surface temperature rises to 1.5 °C.

**Table 1:** The corresponding time in 17 models when global surface temperature rises to 1.5 °C and 2 °C relative to pre-industrial (1861–1880) under RCPs.

Model	1.5 °C			2 °C		
	RCP2.6	RCP4.5	RCP8.5	RCP2.6	RCP4.5	RCP8.5
BCC-CSM1.1	2023/2026	2022/2025	2019/2026	N/N	2040/2050	2038/2038
BCC-CSM1.1(m)	2014/2018	2013/2015	2011/2014	2029/N	2034/2037	2029/2029
BNU-ESM	2007/2012	2006/2017	2009/2016	2025/2031	2022/2031	2024/2024
CanESM2	2017/2017	2020/2020	2009/2015	2033/2033	2030/2035	2027/2030
CNRM-CM5	2040/2045	2035/2038	2030/2030	N/N	2055/2060	2045/2045
CSIRO-Mk3.6.0	2029/2029	2026/2031	2022/2030	2069/N	2043/2053	2043/2043
HadGEM2-ES	2022/2028	2031/2031	2023/2030	2048/N	2043/2045	2036/2038
IPSL-CM5A-LR	2016/2021	2014/2018	2015/2015	2033/2038	2032/2032	2030/2030
IPSL-CM5A-MR	2012/2021	2023/2023	2010/2015	2042/N	2034/2034	2030/2030
MIROC-ESM	2021/2021	2021/2021	2021/2021	2040/2043	2034/2034	2031/2031
MIROC-ESM**-**CHEM	2015/2015	2023/2023	2019/2019	2031/2034	2038/2038	2027/2027
MIROC5	2047/2052	2033/2052	2028/2033	N/N	2055/2058	2043/2052
GISS-E2-H	2025/2042	2023/2023	2021/2026	N/N	2050/2054	2039/2042
CCSM4	2021/2023	2014/2024	2018/2021	N/N	2043/2047	2033/2033
CESM1(CAM5)	2029/2037	2030/2033	2029/2033	2061/N	2047/2053	2043/2043
GFDL-CM3	2021/2036	2025/2033	2026/2029	2052/2059	2040/2040	2041/2043
NorESM1-M	2082/N	2040/2051	2035/2035	N/N	2079/2089	2051/2053
MME	2023/2027	2026/2026	2023/2023	N/N	2043/2046	2037/2037

Note: N indicates events will not happen in 21^st^, the notation/indicates the first time and for 5a reached to a certain temperature threshold.

### Permafrost changes during the 21st century

**Figure 2:**
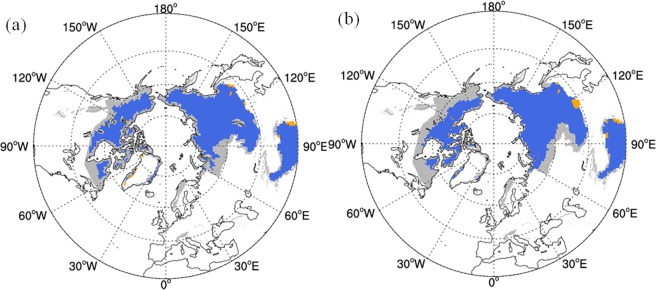
The permafrost extent in Northern Hemisphere during the period of 1986–2005 estimated by Kudryavtsev method (**a**) reanalysis datasets (**b**) CMIP5 models. (Gray region represent the observed permafrost extent from Brown *et al*.^44^; Orange region represent the region where permafrost is overestimated by CLM4.5) (The NCAR Command Language (Version 6.3.0) [Software]. (2016). Boulder, Colorado: UCAR/NCAR/CISL/TDD. http://dx.doi.org/10.5065/D6WD3XH5).

Figure 2 shows the present (1986–2005) permafrost extent estimated from the reanalysis data and the MME of the 17 CMIP5 models. Both estimations are in good agreement with the International Permafrost Association (IPA) data in most regions^48^. Discrepancies between simulated and observed permafrost distributions are mainly located in northern Europe, southern west Siberia and the southern permafrost boundary in North America. The two datasets underestimate the permafrost area in western Siberia and the northern margin of Europe but overestimate the permafrost area on the southern Qinghai–Tibet Plateau and in northern Mongolia. Comparing the permafrost extent estimated by the two datasets, the estimations based on the MME showed a better performance in the northern islands of North America than the estimations based on the reanalysis data. However, the estimations based on the MME underestimate the permafrost in southern North America, which may be related to the difference in permafrost extent between the two datasets. The permafrost areas in the NH estimated from the reanalysis datasets and climate model data are about 15.61 × 10^6^ and 16.24 × 10^6^ km^2^, respectively. The latter is closer to IPA’s estimate of 16.2 × 10^6^ km^2^. However, because the 16.2 × 10^6^ km^2^ figure excludes isolated and sporadic permafrost, the actual permafrost area in the NH is likely to be greater than 16.2 × 10^6^ km^2^.

**Figure 3:**
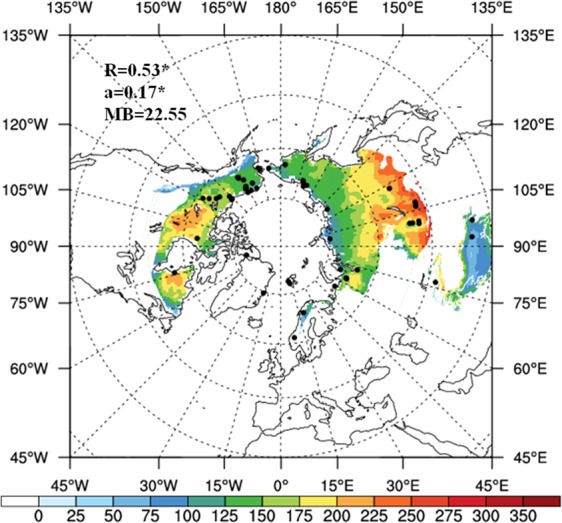
The distribution of ALT (unit: cm) in permafrost region over NH during 1986–2005 simulated by Kudryavtsev method. The black dot represents the 52 observations from GTN-P. ‘R’ is correlation coefficient; ‘a’ is regression coefficient and ‘MB’ represents mean bias between simulation and observation. (The NCAR Command Language (Version 6.3.0) [Software]. (2016). Boulder, Colorado: UCAR/NCAR/CISL/TDD. http://dx.doi.org/10.5065/D6WD3XH5).

Figure 3 shows the active layer thickness (ALT) in NH permafrost regions during the 1986–2005 period, estimated from CMIP5 data. Results indicate that the ALT deepens from high latitude to low latitude and that deeper ALTs (e.g. >225 cm) are mainly located in the Mongolian Plateau and Greater Khingan. Considering the calculation of mean bias, correlation coefficient and regression coefficient under different ALT estimation methods, the Kudryavtsev method could well reflect the correlation between simulation and observation. The correlation coefficient and regression coefficient, both statistically significant at the *p* < 0.001 level, are 0.53 and 0.17, respectively. The mean bias in the Kudryavtsev method is relatively small (23.55 cm), implying the high performance of the Kudryavtsev method for permafrost simulation.

**Figure 4:**
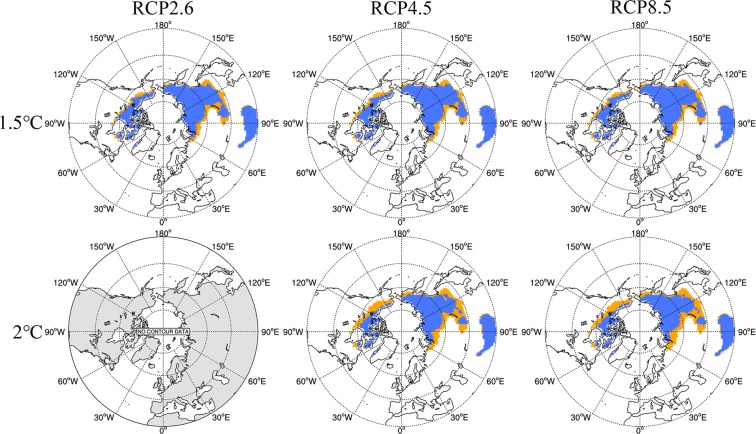
The extent of permafrost in Northern Hemisphere when global average surface temperature rises to 1.5 and 2 °C above pre-industrial (1861–1880) under RCPs. (The orange and blue shaded regions represent the permafrost extent in 1986–2005 and the period corresponding to the certain threshold, respectively). (The NCAR Command Language (Version 6.3.0) [Software]. (2016). Boulder, Colorado: UCAR/NCAR/CISL/TDD. http://dx.doi.org/10.5065/D6WD3XH5).

The response of the components of the cryosphere to global warming is a slow process with gradients^49,50^ and hysteresis. We define the initial year of five consecutive years when global mean surface temperature meets thresholds of 1.5 °C and 2.0 °C, then the subsequent 10 years are regarded as the period that meets the thresholds of 1.5 °C and 2.0 °C. Figure 4 shows the projected change in permafrost extent under the three RCP scenarios for temperature rises of 1.5 °C and 2.0 °C compared to pre-industrial levels. The southern permafrost boundary shifts northwards by 1° to 4° latitude under the three RCPs between the 1.5 °C and 2 °C levels. Compared with the historical period (1986–2005), significant degradation occurs in northern Mongolia and southern Russia. When the global mean surface temperature reaches the 1.5 °C threshold, the MME (Table 2) shows that the permafrost extent in the NH would be 12.81 ± 3.31 × 10^6^, 12.33 ± 4.46 × 10^6^ and 12.09 ± 3.31 × 10^6^ km^2^, respectively, under RCP2.6 (2027–2036), RCP4.5 (2026–2035) and RCP8.5 (2023–2032). Relative to the historical period, these represent reductions in permafrost extent of 23.58%, 24.10% and 25.55%, respectively. However, uncertainty is evident because of the large discrepancies among the models’ performance on temperature simulation over cold regions. When the global mean surface temperature reaches the 2 °C threshold, permafrost areas in the NH would be 10.38 × 10^6^ and 10.13 × 10^6^ km^2^, respectively, in RCP4.5 (2046–2055) and RCP8.5 (2037–2046). These would, respectively, constitute reductions relative to the historical period of 36.08% and 37.62%, respectively, for RCP4.5 and RCP8.5. Compared to the historical period, the most obvious degradation occurs in northern Mongolia and southern Russia, and the southern permafrost boundary moves northwards by about 3° to 4° between the 1.5 °C and 2 °C thresholds under RCP4.5. It must be noted that the global mean surface temperature under RCP2.6 does not rise by 2 °C until the end of the 21^st^ century. The extent of the permafrost in the NH in 2100 is about 12.65 × 10^6^ km^2^, a reduction of 22.11% relative to the historical period. This reduction shows the southern permafrost boundary in Canada and the United States to move northwards and the area of the permafrost in northern Mongolia to decrease slightly. The projected reductions in permafrost area under the three RCPs vary between 23% and 26% at the 1.5 °C threshold and range from 36% to 38% at the 2.0 °C threshold. Relative to the 1.5 °C threshold, the permafrost area at the 2 °C threshold would be reduced by 1.95 × 10^6^ km^2^ (i.e. 15.8%) and 1.96 × 10^6^ km^2^ (i.e. 16.2%), respectively, under RCP4.5 and RCP8.5.

**Table 2:** The permafrost area, absolute change (AC) and relative change (RC) in Northern Hemisphere under global warming.

Region	Type	1.5 °C			2 °C		
		RCP2.6	RCP4.5	RCP8.5	RCP2.6	RCP4.5	RCP8.5
Northern Hemisphere (16.24)	Area (×10^6^ km^2^)	12.81 ± 3.2	12.33 ± 3.2	12.09 ± 2.9	12.65	10.38	10.13
	AC (×10^6^ km^2^)	3.43 ± 3.2	3.91 ± 3.2	4.15 ± 2.9	3.59	5.86	6.11
	RC (%)	21.12 ± 17.8	24.1 ± 17.6	25.55 ± 15.9	22.11	36.08	37.62

Note: In RCP2.6, the global average surface temperature will not reach the 2 °C. 12.64 is the permafrost area in Northern Hemisphere in 2100. The value in bracket represents the decreased permafrost area relative to 1986–2005.

## Discussion

This study used data from 17 CMIP5 models to identify the period corresponding to thresholds of 1.5 °C and 2 °C rises in temperature above pre-industrial levels and estimated the permafrost area and rate of reduction in the NH using the Kudryavtsev method under present and future climate scenarios. The main conclusions of this study are presented below.

The MME analysis showed that the year in which the 1.5 °C rise in global mean surface temperature would be reached would be 2027, 2026 and 2023, respectively, under RCP2.6, RCP4.5 and RCP8.5. However, under RCP2.6, the global mean surface temperature would not reach the 2 °C threshold during the 21^st^ century, reaching only about 1.9 °C by 2100. Under RCP4.5 and RCP8.5, the 2 °C threshold is reached around the middle of the 21^st^ century (2046 and 2037, respectively). Low radiative forcing would slow the temperature increase.

Under continuous global warming from the 1.5 °C threshold to the 2 °C threshold, the projected southern permafrost boundary shifts 1° to 4° northwards under three RCPs. Compared with the historical period (1986–2005), the most significant degradation occurs in northern Mongolia and southern Russia. Under RCP2.6, the extent of permafrost remains almost stablely existed. When the global mean surface temperature reaches the 1.5 °C threshold, the projected permafrost area in the Northern Hemisphere is about 12.81 × 10^6^ km^2^. By the end of the 21^st^ century, the permafrost extent in the Northern Hemisphere is about 12.65 × 10^6^ km^2^. If the global economy keeps increasing at the current rate (e.g. under RCP4.5), the permafrost areas corresponding to the 1.5 °C and 2 °C thresholds, respectively, are 12.33 × 10^6^ and 10.38 × 10^6^ km^2^. This represents a decrease of 1.95 × 10^6^ km^2^, or 15.8%, between the 1.5 °C and 2 °C thresholds. If human society does not take any action to decrease emissions (e.g. under RCP8.5), the degradation of the permafrost is more significant, showing permafrost areas corresponding to the 1.5 °C and 2 °C thresholds, respectively, of 12.09 × 10^6^ and 10.13 × 10^6^ km^2^. This represents a decrease of 1.96 × 10^6^ km^2^, or about 16.2%, between the 1.5 °C and 2 °C thresholds. Relative to the historical period (1986–2005), the permafrost area at the 1.5 °C threshold decreases by 23.58%, 24.1% and 25.55% under the three RCPs. For the 2 °C threshold, increased radiative forcing leads to a greater decline in permafrost area, showing decreases of 22.11%, 36.08% and 37.62%, respectively, under RCP2.6, RCP4.5 and RCP8.5.

It is inevitable that the reduction in permafrost area will lead to a change in surface energy balance and consequently to changes in land–atmosphere interactions that will probably generate ongoing environmental deterioration and unpredictable changes to the climate. However, the results of this study seem slightly hopeful for the permafrost environment, since two-thirds of permafrost will remain under RCP8.5 (2037–2046) at a threshold of 2 °C rise, some permafrost will even remain in some of the Earth’s colder areas until the end of the 21^st^ century^30,43,51,52^. It should be noted that the results of this study do not imply that regions of seasonal frozen ground will expand because of higher winter temperatures. Moreover, although we adopted the results of MME and considered vegetation and soil moisture variability, uncertainties still remain, such as the use of CMIP5 data and lack of precise observations of the soil type and organic matter.

## Methods

This study was based on monthly-mean data from reanalysis projects and the suite of CMIP5 climate models, and only the NH was considered. Owing to the different resolutions of the models and reanalysis data, a bilinear interpolation was used to convert the reanalysis and model datasets to a common resolution of 1° × 1° (latitude × longitude). Monthly precipitation data from the GPCC Full Data Product Version 4, monthly air temperature from the NCEP/NCAR reanalysis dataset and monthly soil moisture to a depth of 10 cm from the Global Land Data Assimilation System (GLDAS) Version 1 were used to estimate permafrost distribution under the present-day climate.

Historical and future simulations from the coupled climate models were obtained from the CMIP5 archive (Table 3). The historical simulations ran from 1850 or 1860 to the end of 2005. We refer to the definition provided by Assessment Report 5 published by the Intergovernmental Panel on Climate Change (IPCC AR5), which designates 1986 to 2005 as a reference period under the current climate^17^. The future simulations used included three different RCPs (RCP2.6, RCP4.5 and RCP8.5), ranging from 2006 to 2100. The model variables include surface temperature, air temperature and precipitation, and the output from run1 of each model was used (e.g. r1i1p1).

**Table 3:** The information of CMIP5 model used in this study (Ensemble means multi-model mean).

Model	Institution	Resolution
BCC-CSM1.1	Beijing Climate Center, China	2.8° × 2.8°
BCC-CSM1.1(m)	Beijing Climate Center, China	1.3° × 1.1°
BNU-ESM	Beijing Normal University. China	2.8° × 2.8°
CanESM2	Canadian Center for Climate Modeling and Analysis, Canada	2.8° × 2.8°
CNRM-CM5	CNRM-CERFACS, France	1.4° × 1.4°
CSIRO-Mk3.6.0	CSIRO Atmospheric Research, Australia	1.875° × 1.875°
HadGEM2-ES	Met Office Hadley Centre, UK	1.875° × 1.25°
IPSL-CM5A-LR	Institut Pierre Simon Laplace, France	3.75° × 1.875°
IPSL-CM5A-MR	Institut Pierre Simon Laplace, France	2.5° × 1.25°
MIROC-ESM	Atmosphere and Ocean Research Institute, Japan	2.8° × 2.8°
MIROC-ESM**-**CHEM	Atmosphere and Ocean Research Institute, Japan	2.8° × 2.8°
MIROC5	Atmosphere and Ocean Research Institute, Japan	1.4° × 1.4°
GISS-E2-H	NASA Goddard Institute for Space Studies, USA	2.5° × 2°
CCSM4	National Center for Atmospheric Research, USA	1.25° × 0.94°
CESM1(CAM5)	NSF-DOE-NCAR, USA	1.25° × 0.94°
GFDL-CM3	Geophysical Fluid Dynamics Laboratory, USA	2.5° × 2.0°
NorESM1-M	Norwegian Climate Center, Norway	2.5° × 1.875°

The extent of the permafrost can be estimated with either indirect methods or direct methods. As indirect methods, we calculated the mean annual air temperature (MAAT), air frost index (F), surface frost index (SFI) and simplified permafrost models (e.g. Kudryavtsev). As direct methods, we diagnosed the temperature of soil layers (TSL) and mean annual ground temperature (MAGT). Although the simulated soil temperature from CMIP5 models or LSMs such as CLM4.5 can also be used in permafrost projection^27,41^, it should be noted that freeze–thaw parameterizations in LSMs might not be perfect. First, most current land surface models are isothermal models and do not consider the interactions between thermal and hydrological processes, thus degrading the simulation performance of the LSM over permafrost regions. This issue and the corresponding parameterization development have been addressed by Wang and Yang^53^ in a recent work. Second, the parameterizations of the occurrence of soil freeze or thaw in current LSMs are not complete^54^, and cannot represent subgrid-scale permafrost distributions (e.g. sporadic and isolated permafrost)^30,55^. Our results^54^ also indicate that the simulations of the freezing–thawing process are not very consistent with observations and would greatly influence the calculation of permafrost extent. Third, since the soil layers in LSM are not uniform and the soil layers are different in different LSMs, it is difficult to directly compare the permafrost extent at the same depth. As a result, interpolation between the soil layers could cause uncertainty in the estimation of permafrost extent. Our preliminary numerical experiment based on CLM4.5 also indicates that CLM4.5 evidently underestimates the permafrost extent (not shown). The Kudryavtsev method is a classic and stable approach in permafrost simulation^56^, which is mainly driven by climate variables (e.g. air temperature, soil moisture and snow depth) and considers the impacts of air temperature, vegetation, snow characteristics, soil texture and thermal properties on the permafrost rather than soil temperature only. Consequently, the Kudryavtsev method was used in this study.

Kudryavtsev’s method assumes that the annual variations of the air temperature can be described by a periodic function. Temperature and its variation amplitude at the soil surface can be expressed as the following three parts:

1 $${\bar{T}}_{s}={\bar{T}}_{a}+{\rm{\Delta }}{T}_{sn}+{\rm{\Delta }}{T}_{veg}$$

2 $${A}_{s}={A}_{a}-{\rm{\Delta }}{A}_{sn}-{\rm{\Delta }}{A}_{veg},$$

where ${\bar{T}}_{a}$ is the MAAT, *A*_*a*_ is the annual amplitude of the air temperature and ${\rm{\Delta }}{T}_{sn}$, ${\rm{\Delta }}{T}_{veg}$, ${\rm{\Delta }}{A}_{sn}$ and ${\rm{\Delta }}{A}_{veg}$ are the adjustments for the thermal effects of snow and vegetation.

The empirical equation accounting for the insulation effect of snow cover has the following form^51^:

3 $${\rm{\Delta }}{T}_{sn}={A}_{a}\{1-\exp [-{Z}_{sn}\cdot {(\frac{\pi {C}_{sn}{\rho }_{sn}}{P\cdot {\lambda }_{sn}})}^{1/2}]\}$$

4 $${\rm{\Delta }}{A}_{sn}=\frac{2}{\pi }{\rm{\Delta }}{T}_{sn},$$

where *Z*_*sn*_ is the snow thickness (m), *λ*_*sn*_ is the snow’s thermal conductivity, *C*_*sn*_ is its specific heat capacity (J kg^−1^ °C^−1^) and ${\rho }_{sn}$ is the snow density (kg m^−3^), which is assumed to be 250 kg m^−3^. The equation for calculating snow depth can be found in Nelson and Outcalt^57^.

The thermal effects of vegetation are represented in the following equations:

5 $${A}_{veg}={A}_{a}-{\rm{\Delta }}{A}_{sn}$$

6 $${\bar{T}}_{veg}={\bar{T}}_{a}+{\rm{\Delta }}{T}_{sn}$$

7 $${\rm{\Delta }}{A}_{veg}=\frac{{\rm{\Delta }}{A}_{1}\cdot {\tau }_{1}+{\rm{\Delta }}{A}_{2}\cdot {\tau }_{2}}{P}$$

8 $${\rm{\Delta }}{T}_{veg}=\frac{{\rm{\Delta }}{A}_{1}\cdot {\tau }_{1}-{\rm{\Delta }}{A}_{2}\cdot {\tau }_{2}}{P}\cdot \frac{\pi }{2},$$

where ${\tau }_{1}$ and ${\tau }_{1}$ are the durations of the cold and warm periods, respectively, and ${\rm{\Delta }}{A}_{1}$ and ${\rm{\Delta }}{A}_{2}$ are the respective differences between the average temperature at the surface and vegetation during the cold and warm periods (°C).

The mean annual temperature at the depth of seasonal thaw ${\bar{T}}_{z}$ was calculated using a semi-empirical equation:

9 $${T}_{num}=0.5\cdot {T}_{s}\cdot ({\lambda }_{f}+{\lambda }_{t})+{A}_{s}\frac{{\lambda }_{f}-{\lambda }_{t}}{\pi }[\frac{{\bar{T}}_{s}}{{A}_{s}}{\arcsin }\frac{{\bar{T}}_{s}}{{A}_{s}}+{(1-\frac{{\pi }^{2}}{{A}_{s}^{2}})}^{1/2}]$$

10 $${\bar{T}}_{z}=\frac{{T}_{num}}{{\lambda }^{\ast }}$$

Here, $${\lambda }^{\ast }=\{\begin{array}{c}{\lambda }_{f},{T}_{num} < 0\\ {\lambda }_{t},{T}_{num} > 0\end{array}$$, *λ*_*t*_ and *λ*_*f*_ are the thermal conductivities of the thawed and frozen soil, respectively (W m^−1^ °C^−1^), and *T*_*s*_ and *A*_*s*_ are the temperature and temperature amplitude, respectively, at the soil surface. When ${\bar{T}}_{z} < 0$, there are permafrost regions, and when ${\bar{T}}_{z} > 0$, there are seasonal frozen soil or non-frozen soil regions.

The following semi-empirical equation for the depth of seasonal thawing or freezing can be applied^51^:

11 $$Z=\tfrac{2({A}_{s}-{\bar{T}}_{z})\cdot {(\tfrac{\lambda \cdot P\cdot C}{\pi })}^{1/2}+\tfrac{(2{A}_{z}C{Z}_{c}+{Q}_{L}{Z}_{c}){Q}_{L}{(\tfrac{\lambda P}{\pi c})}^{1/2}}{2{A}_{z}C{Z}_{c}+{Q}_{L}{Z}_{c}+(2{A}_{z}C+{Q}_{L}){(\tfrac{\lambda P}{\pi C})}^{1/2}}}{2{A}_{z}C+{Q}_{L}},$$

where

12 $${Z}_{c}=\frac{2({A}_{s}-{\bar{T}}_{z})\sqrt{\frac{\lambda PC}{\pi }}}{2{A}_{z}C+{Q}_{L}}$$

13 $${A}_{z}=\frac{{A}_{s}-{\bar{T}}_{z}}{\mathrm{ln}(\frac{{A}_{s}+{Q}_{L}\,/\,2C}{{\bar{T}}_{z}+{Q}_{L}\,/\,2C})}-\frac{{Q}_{L}}{2C},$$

where *λ* and *C* are, respectively, the thermal conductivity (W m^−1^ °C^−1^) and volumetric heat capacity (W m^−3^ °C^−1^) of the soil. *Q*_*L*_ is the latent heat of phase change (J m^−3^). The volumetric heat capacity for soil can be found in de Vries^58^.

Soil type was classified into sand, clay or organic. The International Geosphere–Biosphere Programme (IGBP) soil dataset^59^ of 4931 soil mapping units and the associated sand and clay content for each soil layer were used to create a mineral soil texture dataset^60^. The soil organic matter data were merged from two sources: the International Soil Reference and Information Centre (ISRIC-WISE)^61^ and the Northern Circumpolar Soil Carbon Database (NCSCD)^62^. Both datasets report carbon down to 1 m depth. Carbon was partitioned across the top seven CLM4 layers (~1 m depth) as in Lawrence and Slater^63^. The soil type and organic data were adapted from the land surface data of the Community Earth System Model (CESM). The soil column was subdivided into 10 layers, with the maximum depth of soil being 3.8019 m at a resolution of 0.23° × 0.31°. This selection implies that the permafrost areas and permafrost loss calculated in this study indicate permafrost extent at depths less than 3.8 m. Organic matter density was calculated based on the weights of the soil column at 10 soil layers. The layer of mineral soil and organic matter was considered to be a homogeneous medium with different thermal properties in the frozen and thawed states. The details of the parameter settings in Kudryavtsev’s method can be found in Animov *et al*.^64^.
